# Nitrate Signaling, Functions, and Regulation of Root System Architecture: Insights from *Arabidopsis thaliana*

**DOI:** 10.3390/genes11060633

**Published:** 2020-06-09

**Authors:** Muhammad Asim, Zia Ullah, Fangzheng Xu, Lulu An, Oluwaseun Olayemi Aluko, Qian Wang, Haobao Liu

**Affiliations:** 1 Key Laboratory of Tobacco Biology and Processing, Ministry of Agriculture, Tobacco Research Institute of Chinese Academy of Agricultural Sciences, Qingdao 266101, China; asim.ktk91@aup.edu.pk (M.A.); zianust512@gmail.com (Z.U.); lulu_an9@163.com (L.A.); aluko.oluseun@gmail.com (O.O.A.); 2 Graduate School of Chinese Academy of Agricultural Sciences, Beijing 100081, China; xufangzheng1234@163.com; 3 Key Laboratory for Tobacco Gene Resources, Tobacco Research Institute of Chinese Academy of Agricultural Sciences, Qingdao 266101, China

**Keywords:** nitrate, nitrate transporters, primary response, phospholipase C, root system architecture, lateral roots, primary roots

## Abstract

Root system architecture (RSA) is required for the acquisition of water and mineral nutrients from the soil. One of the essential nutrients, nitrate (NO_3_^−^), is sensed and transported by nitrate transporters *NRT1.1* and *NRT2.1* in the plants. Nitrate transporter 1.1 (*NRT1.1*) is a dual-affinity nitrate transporter phosphorylated at the T101 residue by calcineurin B-like interacting protein kinase (CIPKs); it also regulates the expression of other key nitrate assimilatory genes. The differential phosphorylation (phosphorylation and dephosphorylation) strategies and underlying Ca^2+^ signaling mechanism of *NRT1.1* stimulate lateral root growth by activating the auxin transport activity and Ca^2+^-ANR1 signaling at the plasma membrane and the endosomes, respectively. NO_3_^−^ additionally functions as a signal molecule that forms a signaling system, which consists of a vast array of transcription factors that control root system architecture that either stimulate or inhibit lateral and primary root development in response to localized and high nitrate (NO_3_^−^), respectively. This review elucidates the so-far identified nitrate transporters, nitrate sensing, signal transduction, and the key roles of nitrate transporters and its downstream transcriptional regulatory network in the primary and lateral root development in *Arabidopsis thaliana* under stress conditions.

## 1. Introduction

Nitrogen significantly influences plant growth and development. Plants adopt numerous strategies to modulate the uptake capacity of their roots to cope with spatial and temporal fluctuations in N availability [1]. In plants, the root architecture adjusts to these environmental fluctuations [2,3] and synchronizes the NO_3_^−^ supply and demand inside the plants by the coordination of the systemic signal required to deal with root NO_3_^−^ acquisition [4].

The regulatory pattern of root NO_3_^−^ uptake simplifies the root transport system in two ways; The first is the rapid uptake after the NO_3_^−^ provision, which requires de novo protein synthesis [5,6], and the other is the root NO_3_^−^ efflux, strongly upregulated by N deficiency or low availability and downregulated by high nitrate supply [7,8]. An important hypothesis arising from the recently identified dissimilar NO_3_^−^ influx and efflux and the low- and high-affinity NO_3_^−^ transporters has revealed that several diverse carrier proteins are involved in the root nitrate (NO_3_^−^) transport system. Studies on *Arabidopsis thaliana* suggest that at least two transporters, *NRT1.1* and *NRT2.1,* are involved in NO_3_^−^ sensing [9].

*NRT1.1* activates four signaling mechanisms [10]. Firstly, the primary nitrate response (PNR) [11], the long-term response of *NRT1.1.* Secondly, this then acts as feedback repression of *NRT2.1* under a high NO_3_^−^ supply [12]. Thirdly, the promotion of lateral root (LR) branching by *NRT1.1* in response to NO_3_^−^, inhibiting the emergence of LR primordia at low NO_3_^−^ availability [13], and finally, the induction of genes at high NO_3_^−^ conditions [10]. After nitrate uptake via NRT1s and NRT2s, the part of the NO_3_^−^ influx into the cell is reduced and thereby assimilated as amino acid through a series of enzymes such as nitrate reductase (NR), nitrite reductase (NiR), glutamine synthesis (GS), and glutamate synthase (GOGAT). These NO_3_^−^-mediated developmental processes are governed by a complex network of kinases and phosphatase [14], influencing the primary nitrate response (PNR) [15]. Further studies on sensitive Ca^2+^ biosensors have revealed that NO_3_^−^ treatment upgrades Ca^2+^ concentration in the cytoplasm and nucleus of the protoplast from the mesophyll cells in the tip, pericycle, and stele of the intact roots. In-gel kinase assays have demonstrated that the activity of protein kinases (CPKs) is stimulated by NO_3_^−^ treatment in protoplasts. Previous studies on protoplasts have distinguished subgroup III of the CPKs as regulators of NO_3_^−^ responsive genes [16]. This further confirms the function of NO_3_^−^ as an important signal that regulates gene expression, plant growth, and development [17].

The contribution of the nitrate transporter signaling pathway in the regulation and patterning of root system architecture (RSA) is momentous. This review discusses the significant milestones in the early response signaling and phosphorylation status of NO_3_^−^ in *Arabidopsis* root, with greater emphasis on the signal transduction pathways that shape the architecture of the root in response to altered NO_3_^−^ supply.

## 2. The Nitrate Signaling Mechanism in RSA

### 2.1. Nitrate as Early Response Sensing

The molecular identification and the functional characterization of the genes encoding the NO_3_^−^ transporters in plants began in the mid-1990s and is still an active field of research [18]. The molecular mechanism of NO_3_^−^ signaling transduction has been discovered in *Arabidopsis.* Nitrate transporter1/peptide transporter family (NRT1/NPF), nitrate transporter 2 (NRT2), chloride channel (CLC), and slowly activating anion channel (SLAC/SLAH) are the four nitrate transporter families that have been characterized in *Arabidopsis* [19].

Nitrate transporter 1.1 (*NRT1.1*), also called *CHL1*/*NPF6.3,* belongs to the NRT1/PTR family (NPF) [20]. As a dual-affinity nitrate transporter, *NRT1.1* functions in both low and high nitrate affinity states [21,22], subsequently controlling root architecture by acting as a potential nitrate sensor [23,24] and triggering nitrate-dependent changes in gene expression. Moreover, its nitrate uptake function regulates the expression of key nitrate assimilatory genes. Its affinity state changes according to the phosphorylation status of the T101 residue [14,25]. *NRT1.1* is capable of triggering independent signaling pathways in response to nitrate in *Arabidopsis* roots. Different *NRT1.1* mutant alleles exhibit distinct responses to nitrate at the transcriptome level as well as the repression of LR development [10]. However, in *NRT1.1* (*CHL1/NPF6.3*), the mutant’s *chl1-9* allele, where proline 292 replaces leucine, shows imperfect NO_3_^−^ affinity but exhibits a biphasic initial NO_3_^−^ response for *NRT2.1* [14]. Different studies have revealed that both *chl1-9* and *chl1-5* (deletion mutant of *NRT1.1*) are identical to the long-term suppression of *NRT2.1* expression and LR development without nitrate [10].

Both the primary and secondary NO_3_^−^ responses accomplished by transcriptomic studies indicate that the Affymetrix ATH1 chip has a significant impact on gene expression within 20 min after NO_3_^−^ treatments. These changes were more apparent in roots than in the shoot, with the root having 1176 affected transcripts and only 183 affected transcripts in the shoot [26]. Another study revealed that after NO_3_^−^ supply in nitrate-starved conditions, the NO_3_^−^ transporters *NRT1.1*, *NRT2.1, NRT2.2,* and *NRT2.4* were stimulated [27].

Hence, an additional sensing system may exist for NO_3_^−^ influx or efflux with distinctive or overlapping signaling functions related to *NRT1.1* [28]. For instance, under low NO_3_^−^ conditions, the CBL-interacting protein kinases 23 (CIPK23) phosphorylates at threonine residue 101 (T101) by toggling *NRT1.1* to a high-affinity nitrate transport system [29]. In the early NO_3_^−^ response system, Ca^2+^ is a versatile signaling modulator in various regulatory pathways [30,31]. Ca^2+^ signaling is associated with NO_3_^−^ responsive regulatory genes in *Arabidopsis* roots [16,32]. One should think of how the calcium signal is being triggered. There are some interesting viewpoints about this inquiry, elucidating that *NRT1.1* is regulated by CIPK/CBL proteins, which are also themselves being regulated by calcium [14]. However, the mechanism behind the toggling of CPKs in the nucleus in response to nitrate is unknown [15].

### 2.2. Nitrate and Protein Kinases

The calcineurin B-like protein kinase, *CIPK8,* is rapidly activated by NO_3_^−^ and downregulated in *chl1-5* mutants. To study the function of *CIPK8*, the two independent T-DNA insertion mutants (*cipk8-1* and *cipk8-2*) were isolated and a reduction in the *cipk8* mutant was apparent via the induction of nitrate-responsive genes *NRT1.1*, *NRT2.1*, *NIA1*, and *NiR.* This then clearly demonstrated that *CIPK8* functions as a positive regulator of the primary NO_3_^−^ response in the low-affinity system [25]. Another protein kinase complex, CIPK23-CBL1/CBL9 (CIPK, CBL-interacting protein kinase; CBL, calcineurin-B-like protein), has been associated with dual-affinity transition changes of *NRT1.1* via phosphorylation [14,33]. Further studies have also shown that *FIP1* (factor interacting with poly (A) polymerase 1) adversely regulates the expression of *CIPK8* and *CIPK23* associated with NO_3_^−^ signaling. In the *fip1* mutant, the increased expression of *CIPK23* may affect NO_3_^−^ uptake and subsequently reduce NO_3_^−^ content. Molecular genetics suggest that *FIP1* and *CPSF30-L* operate similar NO_3_^−^ signaling pathways. *FIP1*-induced NO_3_^−^ signaling interacts with *CPSF30-L* and is regulated by *CIPK8* and *CIPK23* [34,35].

The role of the subgroup III protein kinases (CPKs) *CPK10*, -*30*, -*32* in NO_3_^−^ regulated root growth was examined [15]. The NO_3_^−^-induced LR primordial density was reduced and LR elongation was significantly hindered in *icpk* [15], thus associating the inhibition of nitrate–CPK-stimulated genes with transcription, metabolism, and transport activities [15]. The activity of the CPKs can be enriched within 10 min in response to nitrate. These CPKs have been distinguished as the primary regulators that coordinate the essential NO_3_^−^ response [15] and modulate various essential cell and metabolic functions instantly triggered by NO_3_^−^ [36,37].

*CPK10* and *CPK30* have also been shown to be associated with the abscisic acid (ABA) responsiveness of the mesophyll protoplasts, which is a promising avenue of research on the coregulation of NO_3_^−^ and ABA pathways. Both have been speculated to contribute to the regulation of the root growth and gene expression [37]. For instance, *ABI2* (ABA-insensitive 2) phosphatase is a fundamental component of the ABA sensing system [38]. Besides the CIPK23–CBL9 complex functioning in the dual affinity transition changes of *NRT1.1*, *ABI2* and *CBL1* also interact with phosphorylated *CIPK23*, which is recognized as an additional segment of this regulation process. NO_3_^−^ sensitivity instigates a rapid increase in the cytoplasmic Ca^2+^ level downstream of *NRT1.1* in a PLC-dependent manner [28].

**Figure 1:**
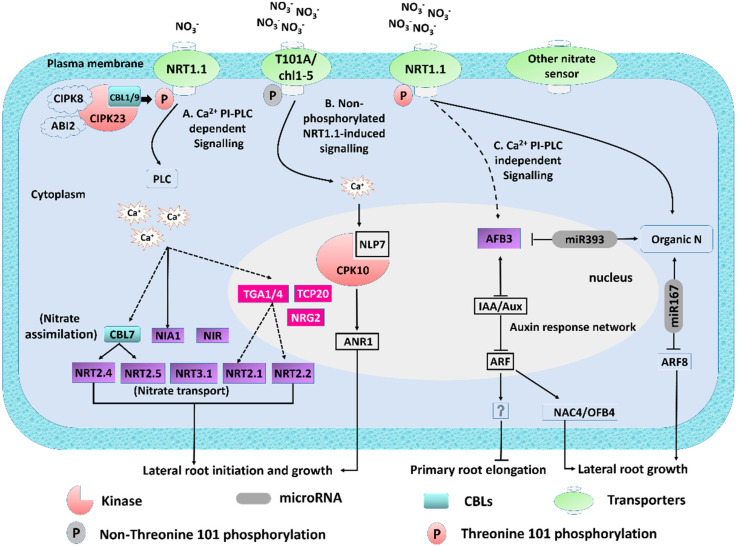
Summary of early responses in nitrate signaling and assimilation. NO_3_^−^ signaling pathway switches its affinity via phosphorylation (modified from Undurraga [41]). Nitrate-responsive genes are depicted in light green, transcription factors in purple, and microRNAs in grey. For clarity purposes, the cell nucleus is shown. Phosphatidylinositol-specific (PI-PLC) and Ca^2+^-dependent pathways. At Low NO_3_^−^ condition, protein kinases CBL1/9–CIPK23 complex phosphorylates *NRT1.1* and changes it into a high-affinity transporter, which activates PLC and results in calcium influx (Ca^2+^ acts as a second messenger). This cascade mediates changes in the expression of transcription factors (TGA1/4 *) and genes involved in nitrate transport (*NRT2.1*, *NRT2.2*, and *NRT3.1*) and nitrate assimilation (*NIA1* and *NiR*). Nonphosphorylated form of NRT1.1-induced signaling. Nitrate-induced Ca^2+^-ANR1 signaling that promotes lateral root (LR) initiation is assumed to be a nonphosphorylated form of *NRT1.1* signaling after the supply of nitrate in limited-nitrate conditions. (**C**) PI-PLC and Ca^2+^-independent pathways. Conversely, *AFB3* is regulated by nitrate in a phospholipase C (PLC)- and calcium-independent manner. *ABF3* modulates the expression of *NAC4* and *OBP4* with subsequent effects on root remodeling. Finally, nitrate assimilation produces organic N, which induces miR393 and represses miR167 (grey) and regulates the abundance of *AFB3* and *ARF8,* respectively. * *TGA1* and *TGA4* are redundant regulatory factors that mediate nitrate responses in *Arabidopsis* roots. However, the interaction between *TGA4* and the PLC–calcium pathway has not been experimentally validated.

In short, nitrate-mediated CPK signaling phosphorylates transcription factors to regulate the expression of downstream genes that affect nitrogen assimilation, carbon/nitrogen metabolism, and proliferation [15]. However, it is possible that additional NO_3_^−^ sensors and *NRT1.1*-independent pathways could be involved in the Ca^2+^ influx and other signaling measures [28,39] (Figure 1). An increase in Ca^2+^ initiates a change in the protein phosphorylation status while controlling the movement of the key component of the NO_3_^−^ signaling pathway. *CPK10*, *30*, and *32* work as regulators of the essential NO_3_^−^ response, linking the Ca^2+^ influx with the phosphorylation of the target proteins. CPK activation could also be linked with NRT1.1-dependent pathways [28].

## 3. Nitrate Signaling and Calcium

Previous studies have revealed that nitrate treatments abruptly raise cytoplasmic Ca^2+^ levels in the roots as well as in the entire seedling [16] (Figure 1). This confirmed that the function of Ca^2+^ in nitrate signaling originates from early research on corn and barley, where EGTA or LaCL_3_ alters the expression of NO_3_^−^-responsive genes. The potential role of Ca^2+^ as a second messenger was thus indicated [32,40].

Ca^2+^ sensor proteins perceive changes in the (Ca^2+^)_cyt_ and subsequently transduce downstream signaling cascades to stimulate alteration of enzymatic activity, cytoskeleton orientation, phosphorylation, and gene expression [42,43]. This was further confirmed by the pretreatment of seedlings with phospholipase C inhibitors or Ca^2+^ channel blockers, which severely affected NO_3_^−^-responsive gene expression in *Arabidopsis,* indicating the function of Ca^2^ as a secondary messenger in NO_3_^−^ signaling pathways. A model was therefore suggested, where the (Ca^2+^)_cyt_ level increases by *NRT1.1* and phospholipase C activity in response to NO_3_^−^, which is required for changes in the prototypical NO_3_^−^-responsive gene expression [16]. Taken together, both *NRT1.1* and phospholipase activity are mandatory for NO_3_^−^- mediated increase in cytoplasmic Ca^2+^ levels and IP3 (Figure 1) [16].

PLC enzymes are membrane-associated, resulting in the remodeling of lipid membranes by the breakdown of phospholipids and the subsequent production of multiple secondary messengers [16]. In plants, two classes of PLCs exist, and they are distinguished based on their substrate specificity. One is phosphatidylinositol-specific (PI-PLC) and the other is non-specific (NPC). Plant NPCs share homology with bacterial PLCs. NPCs can incline either phosphatidylcholine-specific phospholipase C (PC-PLC), phosphatidylethanolamine (PE-PLC), or phosphatidylserine (PS-PLC). However, PI-PLC is the most considered class of PLC, which hydrolyzes phosphatidylinositol 4, 5-bisphosphate (PIP2) from the plasma membrane to create IP3 and diacylglycerol (DAG) [44]. The nitrate signaling and phosphatidylinositol-specific PI-PLC links were found in *Arabidopsis*. Nitrate triggers Ca^2+^ and inositol 1, 4, 5- triphosphate (IP3), which were not witnessed in the plant’s pretreatment with PLC inhibitor U73122. For instance, the *NRT1.1* mutants, *chl1* and *chl9*, revealed that this was an *NRT1.1*-based response. The associated rise in IP3 after NO_3_^−^ treatment also suggested that the activity of phospholipase C (PLC) was associated with this signaling pathway [16].

In *Arabidopsis thaliana*, expression analysis of different PI-PLC genes demonstrated that PLC isoforms were differentially expressed in different plant organs [45,46,47] and that the expression of *AtPLC1*, *2*, *3*, *4*, *5,* and *9* were root-specific [48,49].

### 3.1. Nitrate-Induced Ca^2+^ and PI-PLC-Dependent Signalling

Phosphatidylinositol-specific phospholipase C (PI-PLC) is the major part of nitrate signaling and transport, modulated by the phosphorylation/dephosphorylating process. Both plasma membrane and tonoplast nitrate transport activity are regulated by phosphorylation [27,29]. In *Arabidopsis*, Ca^2+^ has a definite role in plant signal transduction and is also significant for the NO_3_^−^-mediated signaling of gene expression. As stated earlier, NO_3_^−^ treatment rapidly increased the cytoplasmic Ca^2+^ level in the roots [27,29] (Figure 1) and nitrate is absorbed in the root cell by plasma-membrane-localized nitrate transporter families, NRT1 and NRT2 [22]. *NRT1.1/CHL1* is a low-affinity transporter that switches to a high-affinity transport system when *NRT1.1* is phosphorylated at the threonine residue 101(T101) by protein kinase CBL1/9-CIPK23 [9]. The protein complex CIPK23–CBL9 (CBL-interacting protein kinase (CIPK); calcineurin-B like protein (CBL)) and *CIPK8* have been implicated in the dual-affinity transition changes of *NRT1.1* through phosphorylation [33]. More recent studies have revealed that a protein phosphatase 2C (PP2C) family member, *ABI2* (ABA-insensitive 2), and the calcium sensor *CBL1* were distinguished as supplementary constituents that modulate *NRT1.1* transport functions and *NRT2.1* expression in root growth NO_3_^−^ responses [38] (Figure 1).

Hence, the phosphorylation activates a weak upregulation of high-affinity nitrate transporter NRT2.1 [14], and subsequently induces *NRT1.1*, *NRT2.1*, *NRT2.2*, and *NRT2.4* under nitrate-starved seedlings after nitrate supply, while upregulating all the nitrate assimilatory genes [27,50]. CPK phosphorylates the NLP TFs, particularly *NLP7,* which interact with *CPK20* in the nucleolus under NO_3_^−^ availability. Besides *NPL7,* more TFs, such as *TCP20,* also contribute to the NO_3_^−^-induced transcriptional changes and systemic signaling. In contrast, *TGA1/4* controls the genes which participate in the PNR, transport, metabolic, and developmental processes [28].

Under limited-nitrate conditions, the *NRT1.1* is, therefore, phosphorylated at the T101 in order to stimulate *NRT1.1* association with membrane microdomains at the plasma membrane (PM). When nitrate supply is increased, the nonphosphorylated *NRT1.1* shows oligomerization and low structural mobility at the PM, thereby initiating rapid inducible endocytosis. These activities could promote LR growth by switching NRT1.1-auxin transport activity on the PM and stimulating Ca^2+^-ANR1 signaling from the endosomes (discussed in detail in Section 3.2.1, nonphosphorylated nitrate signaling) [51].

### 3.2. Differential Phosphorylation State of Nitrate Transporters

*NRT2.1* is firmly induced by the nonphosphorylated form of *NRT1.1*, which transports NO_3_^−^ at low affinity. After prolonged exposure to NO_3_^−^ treatment, *NRT2.1* is repressed by phosphorylated *NRT1.1*. The NO_3_^−^ transport capacity under this condition remains obscure [52].

**Figure 2:**
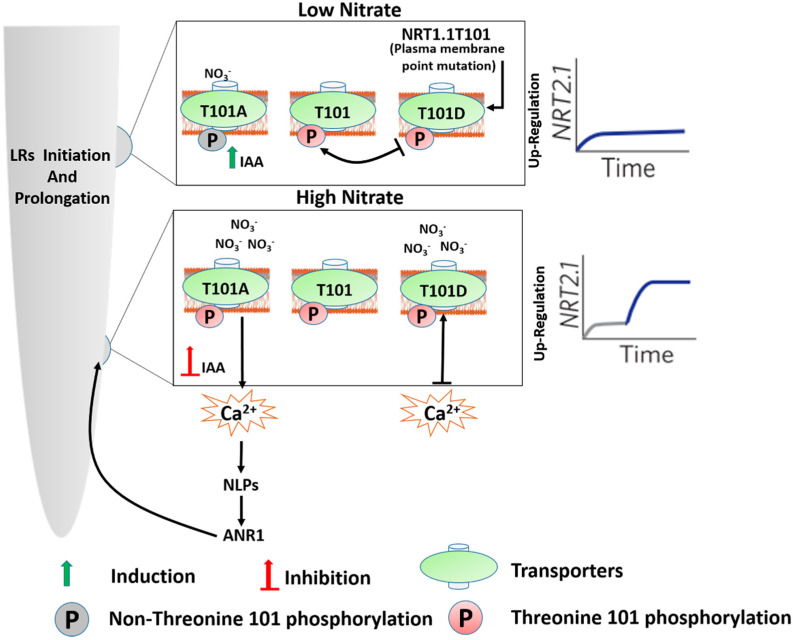
The schematic diagram describes the differential phosphorylation status of *NRT1.1.1T101* at *plasma membrane (PM)* in the *Arabidopsis* root, modified by [52]. The layout represents the two binding sites’ low affinity (LA) and high affinity (HA) of *T101A*. The *T101A* mutant at the LA binding site follows the *NRT1.1-ANR1* signaling pathway upon prolonged exposure to the NO_3_^−^ under low-nitrate conditions, resulting in LR elongation. This is a nonphosphorylated form of NRT1.1-induced signaling that promotes LRs. In the inserted graph, the grey line represents the weak upregulation of *NRT2.1* under low nitrate, and the blue line represents the strong upregulation of *NRT2.1* under high nitrate. The graphs on the left and right represent the *NRT2.1* induction; see text for more details.

However, after the point mutation at the plasma membrane, the mode of *NRT1.1T101* phosphorylation may be different in both NO_3_^−^ uptake and signaling. Transgenic plants of *T101A*, which mimic the *NRT1.1/CHL1* dephosphorylation, exhibits only low-affinity NO_3_^−^ uptake, but can also sense NO_3_^−^ at high-affinity range, with the high-affinity for NO_3_^−^ being comparatively less than the wild-type (WT) [14]. These properties propose that WT *NRT1.1* and the *T101A* mutant may have two NO_3_^−^-binding sites; high affinity and low affinity. It is worth noting that only the low-affinity binding site of the *T101A* mutant can be transported over the plasma membrane (PM). Unlike NO_3_^−^ uptake, NO_3_^−^ binding to both sites of *T101A* mutants could trigger the NO_3_^−^ response. This could justify the reason why the *CHL1T101A* mutant still exhibits a biphasic primary response [14]. In contrast, *T101D-* expressing transgenic plants that mimic phosphorylated *NRT1.1/CHL1* displayed only high-affinity NO_3_^−^ uptake activity and are activated only at a high-affinity primary NO_3_^−^ response. This suggests that *T101D* can only bind NO_3_^−^ with a high-affinity uptake system [14]. Subsequently, it could be possible that binding sites with low affinity could be blocked by T101 phosphorylation [14] (Figure 2).

The two NO_3_^−^ binding sites depicted here (Figure 2) have two adaptations of a single binding site. Taken together, these findings suggest that at the low level of NO_3_^−^ sensing, T101 phosphorylation keeps the PNR, whereas, for uptake and substrate-binding, T101 phosphorylation may repress the low-affinity NO_3_^−^ binding and is then required to use the high-affinity transport system [14].

#### 3.2.1. Non-Phosphorylating Form of NO_3_^−^-Induced Signaling

*NRT1.1* contributes to the NO_3_^−^-mediated auxin transport, regulates auxin storage, and subsequently influences LR development [53]. The signaling network comprising of Ca^2+^, Ca^2+^-protein kinases (CPKs), and NIN-like protein (*NLPs*) interacts with NO_3_^−^ via primary transcription to regulate LR growth [23,45]. In addition to this Ca^2+^-, ARABIDOPSIS NITRATE REGULATED1 (*ANR1*), a transcription factor functioning downstream of *NRT1.1* and *NLP7,* has been involved in LR elongation under high NO_3_^−^ (HN) conditions [54]. In a plant developmental network, nitrate-induced Ca^2+^-ANR1 signaling is a nonphosphorylated form of *NRT1.1* signaling, promoting LR growth. NO_3_^−^ triggers a unique Ca^2+^-CPKs-NLPs signal, acting as downstream segments of *NLP* and *ANR1*, subsequently controlling LR elongation [16] (Figure 1).

*NRT1.1* phosphorylation influences cytoplasmic Ca^2+^ ((Ca^2+^)_cyt_) levels in the epidermal cells of the LRs, which was measured by using Fluo-4 dye in various genotypes [52]. In view of the pseudocolor and kymograph pictures of wild-types (WTs), after NO_3_^−^ stimulation, (Ca^2+^)_cyt_ signaling was screened at the proposed 60-second period. This was previously depicted by [16]. The researcher found that NO_3_^−^ explicitly induced Ca^2+^ signature in the WT but not in *chl1-5* mutant seedlings. Under both HN and LN conditions, *T101A* seedlings exhibited a transient increase in (Ca^2+^)_cyt_ [51], while *T101D* seedlings displayed a decrease in [Ca^2+^]_cyt_. Concomitantly with [Ca^2+^]_cyt_ accumulation, HN-stimulated expression of *ANR1* in LRs is sensed in *T101A,* but not in *T101D*. In the light of these findings, it is suggested that a nonphosphorylated form of *NRT1.1* could activate the Ca^2+^-CPKs-NLPs signaling pathway to induce the expression of *ANR1*, and subsequently control LR elongation [51]. It was analyzed that intracellular transport of *T101A* and *T101D* in LR cells showed that differential phosphorylation of *NRT1.1* enhanced the implementation of *NRT1.1*-stimulated signal transduction in LR growth [51]. Phosphorylated *NRT1.1* takes up the sparingly accessible NO_3_^−^ from the soil at high affinity and induces the *NRT2.1* expression to a lower extent compared to the low-affinity state [52] (Figure 2). Under high NO_3_ conditions, NRT1.1-induced auxin transport is inhibited, and shortly after NO_3_^−^-treatment, the dual affinity modes of the *NRT1.1* are regulated at Thr-101(T101) phosphorylation [52].

As mentioned earlier, under low NO_3_^−^ conditions, phosphorylation at T101 stimulates *NRT1.1* association with a functional membrane microdomain at PM [51], confirming the NRT1.1-mediated auxin flux, and subsequently repressing their growth by reducing the LRP auxin level. With an increased NO_3_^−^ level, nonphosphorylated *NRT1.1* shows oligomerization and low lateral mobility at the PM and rapid inducible endocytosis. This activity may stimulate LR development by supporting NRT1.1-auxin transport activity on the PM to induce Ca^2+^-ANR1-signaling from the endosome [51]. Further studies have shown that seedlings of *T101A* had much higher LR density than that of *T101D* when grown under low NO_3_^−^ conditions (0.2 mM), whereas in high NO_3_^−^ conditions (1 mM), no significant difference was observed in the LR density of the mutants compared to WT plants [51]. These findings confirm that that *T101A* and more nonphosphorylated WT *NRT1.1* promote LR growth in LN by suppressing basipetal auxin transport, and subsequently accumulating auxin in the LR tips [51].

### 3.3. Nitrate-Induced Ca^2+^ and PI-PLC-Independent Signaling

Ca^2+^ and PI-PLC are not affected by the expression of NO_3_^−^ responsive auxin signaling F-Box3 (AFB3) protein, indicating that beyond Ca^2+^ and PI-PLC, there is a PI-PLC-independent pathway that controls the regulation of the nitrate-sensitive genes [16,55] (Figure 1). Hence, *NRT1.1* toggles within the phosphorylation status of a critical threonine residue from low- to high-affinity states. This residue is amongst the second and third transmembrane helices of NRT1.1 located in the intracellular side [14,25].

In *Arabidopsis* root, Ca^2+^ and PI-PLC-independent miR393/AFB3 regulatory modules are recognized as nitrate responsive genes, which assimilate nitrate and auxin signaling [56]. Nitrate induced LRs are dependent on miR167, and its target auxin-responsive factor *ARF8* mRNA [57] plays a distinctive role in regulating several genes connected via a network to promote the stimulation of LR initiation and inhibition of elongated roots in response to N [57] (Figure 1). This earlier identified regulatory module, controlled by miR393 microRNA and the *AFB3* auxin receptor, stimulates LRs in response to external and internal NO_3_^−^ applications [51,58]. *AFB3* is induced by NO_3_^−^ and repressed by miR393, whereas nitrate reduction and assimilation produced N metabolites, which induces miR393 [59] (Figure 1). Furthermore, *AFB3* coregulates *NAC4* and *OBP4*, and this coregulation is confirmed by using the green fluorescence protein (GFP)-expressing lines after 2 h, in response to nitrate. *AFB3,* activated in the pericycle, indicated that the *AFB3-NAC4-OBF4* complex might build a regulatory module that controls LR growth in a NO_3_^−^-dependent manner [56].

Nitrate-stimulated *AFB3* induced in the root might be a specific signaling network of Aux/IAA and *ARF* factors to modulate *NAC4* activation and LR growth. The abundant Aux/IAA-ARF modules chronologically generate new LRs and control LR development in *Arabidopsis*. The lateral root basal meristem (the zone between meristem and elongation) depends on IAA28 and ARF proteins, which include transcription factors *ARF5*, *ARF6*, *ARF7*, *ARF8,* and *ARF19* [13,53]. In plant RSA, the LR initiation and emergence of the *AFB3* overexpression line and the *afb3* mutant line have emerging roles compared to wild-types and display increased growth of LRs under nitrate-sufficient conditions. Additional findings revealed that the transcription factor *NAC4*, which functions downstream of *AFB3,* might be involved in two dependent pathways of RSA regulation [52,58]. Following *AFB3*, *NAC4* acts downstream in the pericycle cell to alter LR density in nitrate treatments [9,51].

#### Auxin Response Network

Auxin signaling is primarily passed over by transcriptional pathways for morphogenesis and developmental processes, which include TRANSPORT INHIBITOR RESPONSE1/AUXIN SIGNALING F-BOX (TIR1/AFB) proteins, AUXIN/INDLOE-3-ACETIC ACID (AUX/IAA) transcriptional co-regulators and AUXIN RESPONSE FACTOR (ARF) transcription factors [60,61,62]. At low levels of auxin concentration, members of the transcriptional inhibitor family AUXIN/IAA-INDUCIBLE (AUX/IAA) interact with the DNA-binding protein of ARF [56,57], while the ARF proteins function to detect the auxin-response promoter elements (AuxREs) in various auxin-regulated genes to activate or suppress their expression [63,64]. AUX/IAA protein inhibits the ARF function either by passively inhibiting ARF proteins from their target promoters [65] or by binding ARF with the corepressor TOPLESS (TPL) for inactivation of the chromatin and silencing of ARF target genes [56,59,66]. An increase in auxin concentration by an auxin-induced module of the coreceptor complex consists of F-box protein from the TRANSPORT INHIBITOR RESPONSE 1 (TIR1)/AUXIN SIGNALING F-BOX PROTEIN (AFB) family and is an Aux/IAA member [60,67,68]. TIR1/ABFs, a subunit of nuclear S-PHASE KINASE ASSOCIATED PROTEIN 1-CULLIN-F-BOX PROTEIN (SCF)-type E3 ubiquitin-protein ligases (SCFTIR/AFB), stimulate the recognition of substrates. The auxin response is initiated by connecting hormones to the TIR1/AFB receptor. The auxin receptor is part of the SCFTIR1/AFB ubiquitin ligase complex [69,70]. Binding of auxin to its receptor TIR1/AFB activates the information and breakdown of the polyubiquitination of the Aux/IAA inhibitor, which subsequently releases the inhibition of ARF transcription factors, which induce the transcription of auxin-responsive genes [71,72]. This represents the pivot of auxin signaling.

In a simpler form, auxin-initiated AUX/IAA removal relieves ARF inhibition and activates the transcription of primary genes. Remarkably, the auxin response network is enough to reconstitute the AuxRE-dependent activation of reporter genes in yeast [73]. Hence, in *Arabidopsis* root, a miR393/*AFB3* regulatory module is recognized as nitrate-responsive, which assimilates nitrate and auxin signaling to promote root growth [56].

## 4. The Effects of Nitrate on RSA

### 4.1. Effects on Primary Root Growth and Development

**Figure 3:**
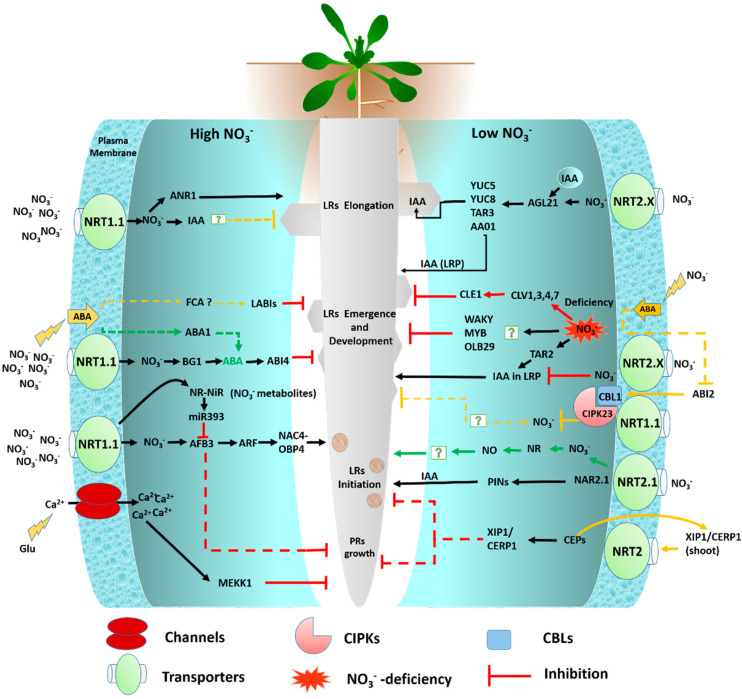
The schematic diagram presents the multiple pathways regulating the root system architecture (RSA; lateral and primary root) response to the localized and high nitrate conditions in *Arabidopsis*. Only those pathways discussed in the present review are depicted. The green arrows indicate systemic transport and assimilation, the black arrows indicate positive signaling as a stimulatory effect, red lines indicate negative signaling as an inhibitory effect, the orange lines depict the unknown positive and negative signaling pathways, and dotted lines represent the unconfirmed nitrate-mediated signaling pathways. The low nitrate and severely low nitrate conditions have been reported to have a stimulatory and inhibitory effect on LR development, respectively, while high NO_3_^−^ supply has an inhibitory effect on LR growth [24] (see text for further information). External NO_3_ regulates primary root growth in *Arabidopsis*. The receptor for the external glutamate signal is shown as a glutamate-gated Ca^2+^ channel because these are known to be activated at root tips [79]. However, its specific role in this signaling pathway is unconfirmed (see text for further information).

Generally, the primary root (PR) growth in *Arabidopsis* is typically found to be relatively insensitive to or even induced by the normal range of NO_3_^−^ concentration [24,74,75]. It could be inhibited under some culture conditions by moderately high NO_3_^−^ supply [56]. It was presumed that *AFB3* controls LR initiation and PR development by two distinct pathways, of which one is NAC3-dependent, while the other is NAC3-independent [76]. However, studies on the effect of amino acid and peptide on root growth and branching have gained little attention [77]. At a low concentration of glutamate (<50 mM), the PR tip has a unique and differential effect on root architecture, inhibiting PR growth and subsequently stimulating LR growth [78] (Figure 3).

To this end, this response is glutamate-specific in *Arabidopsis* since an ongoing study of the impact of 17 other proteinogenic amino acids on the architecture of the roots found none that could produce its distinctive effect on root architecture [77]. By using a chemical genetic approach, the *MEKK1 MAP* kinase gene has since been investigated as part of the glutamate signaling pathways in PR tips [80]. *MEKK1* functions mainly as a distinctive immune system and its expression was demonstrated to be profoundly receptive to a variety of abiotic factors [81]. Nitrate exhibits a strong signal to stimulate the primary root development by enhancing the activity of the meristem and cytokinin signaling. Cytokinin sensing and biosynthesis mutants showed shorter roots compared with wild-type when subjected to NO_3_^−^ treatments, especially when NO_3_^−^ is the primary source [82]. Histological studies of the root tip revealed reduced cell division and elongation in the cytokinin receptor double mutant *ahk2/ahk4* (histidine kinase) compared with WT plants under adequate NO_3_^−^ supply. It is worth noting that as NO_3_^−^-mediated restriction in the root growth was observed between 5 and 6 days after planting, the WT plants had the potential to recover from the growth-restricted condition, whereas cytokinin signaling or biosynthesis mutants were most certainly not capable of recovering [82].

In addition, the transcriptomic analysis indicated that genes associated with both cell division and elongation are possibly significant for PR development in response to NO_3_^−^, thereby indicating the interaction between nitrate and cytokinin signals in regulating PR development in *Arabidopsis* [82].

### 4.2. Effects on Lateral Root Growth and Development

The growth of lateral roots is strongly affected by the concentration of N in the growth environment. For instance, in low NO_3_^−^ soil, patches of high NO_3_^−^ have a localized stimulatory impact on LR development, which varies in different plant species [2,74], whereas under high NO_3_^−^ conditions (with no restricted growth), LR development is repressed [83]. Further studies also revealed that NO_3_^−^ plays a prominent role in regulating LRs. Generally, low NO_3_^−^ has a dual effect on the LRs, such as stimulatory as well as inhibitory effects, whereas high NO_3_^−^ supply only exhibits an inhibitory effect on LR growth and development of LRs [4]. In other words, there are two clear morphological adaptations. Under N-deficient conditions, the LRs are significantly stimulated; however, when exposed to more severe N deficiency, the entire LR length reduces and LR formation disappears [13]. This is initiated by the signaling impact of NO_3_^−^ itself, rather than downstream metabolites [2].

#### 4.2.1. Stimulatory Effect of Low Nitrate on LR Growth

The low NO_3_^−^-stimulated *Arabidopsis* LR development depends on the role of the auxin biosynthetic gene *TAR2* (tryptophan aminotransferase related 2; Figure 3), which is expressed in the pericycle and vasculature of developed roots close to the root tip and is stimulated under low-nitrogen conditions. In WT plants, the low NO_3_^−^ restored auxin accumulation in the primordial of the nonemerged LRs, with an additional three cell layers and LR emergence. On the other hand, these low N-stimulated auxin accumulation and root developmental responses were disrupted in *tar2* null mutants [4,51]. Subsequently, *TAR2* is required for restructuring the root architecture in response to low N conditions. Another nitrate responsive gene, *BBX16* (bobby sox homolog), belongs to the constans-like zinc finger family. The *bbxl16-1* mutant affects lateral root length (LRL) in response to NO_3_^−^, with longer LRs by 1 mM KNO_3_^−^ as low nitrate treatment. The *bbx16-1* mutants produce larger LRs under NO_3_^−^ limitation [48] (Table 1).

When the NO_3_^−^-deficient condition becomes severe, the *Arabidopsis* AGL17-clad MADs-box gene *AGL21* is induced by N shortage and auxin to promote LRs in *Arabidopsis*, whereas *agl21* mutants exhibit a reduction in LR elongation in response to low NO_3_^−^ treatments. Furthermore, the auxin biosynthesis genes *YUC5*, *YUC8,* and *TAR3* are significantly upregulated in overexpressing (OE) lines and downregulated in *agl21* mutants, demonstrating that *AGL21* enhances the local auxin activity in the LR primordial, and thus substantially influencing LR growth regulation [77,80,88].

Previous studies about rice have revealed that the *AtNRT2.1* homolog *OsNAR2.1* knock-out mutant initiates the inhibition of LRs under low NO_3_^−^ concentration by reducing PIN protein levels in the roots [89]. *NRT2.1* positively regulates LRs by influencing the polar transport of auxin under low NO_3_^−^ conditions. The impact of *NRT2.1* on LR growth is possible by a combination of NO_3_^−^ uptake and signaling. *NRT2.1* cannot function independently as a NO_3_^−^ transporter. Hence, *NRT2.1* might act as a key factor in this signaling pathway [4]. It was thus demonstrated that *OsNRT2.1* could be involved in the nitrate-dependent pathway of root elongation by regulating auxin transport to the roots under low NO_3_^−^ conditions [90]. Apart from the aforementioned pathways comprising both transcriptional factors and hormonal signals, nitric oxides (NOs) have been accounted for as a significant NO_3_^−^-mediated signal which regulates RSA in plants [79,82]. In rice, NO produced by NR could enhance the inadequate production of N by developing LR initiation under partial NO_3_^−^ availability [91,92]. To this end, LRs are significantly stimulated by mild NO_3_^−^ deficiency. Different molecular players are involved in the regulation of different stages of plant growth and development.

#### 4.2.2. Inhibitory Effects of Severely Low Nitrate on LR Growth

Earlier studies have found that the impact of NO_3_^−^ was related to the ability of the localized NO_3_^−^ supply to stimulate LR elongation [23,83]. Experimental estimation of using a limited, rather than uniform, NO_3_^−^ treatment initiates the specific effects of the external NO_3_^−^ on LR development, and this can be observed under conditions where the systemic effects, due to changes in the N status of the plant, can be limited to a greater extent [2,4]. Under severe N deficiency, both LR formation and length are repressed in plants [93].

A recent investigation [4] featured the vital role of the peptide-receptor signaling module, which comprises N-responsive CLE (CLV3/ENDOSPERM SURROUNDING REGION (ESR))-related peptides and the CLAVATA1 (CLV1) leucine-rich repeat receptor-like kinase regulatory module, in regulating LR growth of *Arabidopsis thaliana*. *CLE1*, −*3,* −*4*, and −*7* are expressed in root pericycle cells of *Arabidopsis* roots. Under NO_3_^−^ deficient conditions, overexpression (OE) of *CLE* genes results in the repression of LR emergence from the PR. This inhibitory action of the CLE peptides also affected LR development required for the feedback function of *CLV1* expressed in the phloem of the root companion cells, indicating that the downstream signal is transmitted via phloem for the systemic regulation of RSA [4]. An additional system, downstream of *CLV1* feedback, regulates the transcript level of the N-responsive CLE genes in the roots for fine-tuning of the signal amplitude [4,89]. In other words, CLEs-CLV acts as a regulatory module in NO_3_^−^ signaling pathways, and it also antagonistically controls the growth of LRs under limited N conditions [4,94].

Similarly, one member of the CEP (C-TERMINALLY ENCODED PEPTIDE) gene family has been shown to arrest root growth [95]. The analysis of OE-lines of several CEP genes demonstrates their distinctive function. It was reported that CEPs have an antagonistic effect on LR growth while initiating a delay in PR and LR growth [95].

Another mechanism of the systemic inhibition of LR growth is associated with the inhibition of LRs in response to NO_3_^−^. Limited NO_3_^−^ supply significantly increases abscisic acid (ABA) accumulation, as this ABA accumulation inactivates its coreceptor *ABI2* (ABA-insensitive 2) and protein phosphate 2C (PP2C) [96] (Figure 3). The *ABI2* then co-interacts with Ca^2+^-sensor subunit *CBL1* and the kinases (CBL1-CIPK23) complexes, with their substrate being *NRT1.1/NPF6.3*. Hence, under low NO_3_^−^ conditions, the protein kinase *CIPK23* phosphorylates *NRT1.1* to sustain movement at low NO_3_^−^ concentrations [14] (Figure 3). This hypothetical pathway, reconfirmed in recent studies, has revealed that alteration in the *ABI2* status promotes the activation of the CBL1–CIPK23 complex, and subsequently reduces root NO_3_^−^ uptake by inhibiting *NRT1.1* transport activity under NO_3_^−^-deficient conditions [38]. However, the downstream constituents of this pathway are still unknown. It is thus still unclear whether the antagonist effect of ABA on the LRs, subjected to low N conditions, is a consequence of the disrupted NO_3_^−^ signaling pathway or physiological function of ABA itself.

Moreover, irrespective of the NO_3_^−^ activity, *NRT2.1* functions as a NO_3_^−^ sensor or signaling component to inhibit LR initiation under low-NO_3_ conditions [23,93]. However, their exact underlying mechanism is still unclear. The negative effect of *NRT1.1/NRT2.1* on LR growth indicates the distinct systemic pathways under limited NO_3_^−^ supply [4]. Taken together, *NRT1.1/NRT2.1* has a negative role in LR growth and possibly clarifies the inhibitory effect of high NO_3_^−^ on L development. *NRT1.1/NRT2.1* functions negatively and also have an inverse effect on these signaling pathways to control LR growth and development under limited NO_3_^−^ conditions. The action of each pathway depends on the level of the N deficiency in plants or their specific ecological conditions [4].

#### 4.2.3. Systemic Inhibitory Effect of High External Nitrate on LR Growth

The LRs of *Arabidopsis* exhibited two different responses to high NO_3_^−^. High NO_3_^−^ (10 mM) conditions decreased the entire root system, whereas, when plants are subjected to low NO_3_^-^ concentrations (10 µM), the PR part exposed to high NO_3_^−^ triggered the local induction of LR elongation [2,83]. However, the global inhibitory effect of NO_3_^−^ appeared to be as a result of prolonged exposure of plants to ample NO_3_^−^ supply. The LR elongation under this condition was also suppressed in the areas of the root system that were subjected to the state of low NO_3_^−^ conditions [2,97].

As reported earlier, the *AFB3* receptor gene is strongly induced by NO_3_^−^, and the LR initiation is specifically diminished in *afb3* mutants [59]. Research on the nitrate reductase (NR)-null mutants has revealed that NO_3_^−^ itself was the main stimulator of *AFB3*. *AFB3* expression feedback is regulated by nitrate-assimilatory products, such as miR393, a micro RNA that targets *AFB3* transcript for degradation. This pathway has further confirmed the findings that nitrate (NO_3_^−^) induced *NAC4* and *OBP4* transcription factors, functioning downstream of *AFB3*. Taken together with the results obtained from *nac4* mutants, the *afb3* mutant displays an apparent reduction in LR growth in response to NO_3_^−^ [56]. Similarly to this was the influence of the *myb29-1* allele on lateral root length (LRL) when subjected to diverse NO_3_^−^ conditions, exhibiting shorter lateral root length (LRL) at high NO_3_^−^ (10 mM KNO_3_^–^) treatments [48]. However, the *rav2-1* and *erf107-1* alleles, which are genotype-dependent, exhibited reduced lateral root length (LRL) when subjected to both 1 and 10 mM KNO_3_ conditions [48] (Table 1). Recent studies have demonstrated that high-affinity NO_3_^−^ transporter *AtNRT2.1* may be involved in the inhibition of LR initiation at high C: N ratios [98]. Also, the involvement of the ABA affecting LR growth, in response to NO_3_^−^, might be connected to the recently identified ABA receptor [98]. Nitrate reductase (NR)-lacking mutants display sensitivity to this systemic inhibitory effect, indicating that NO_3_^−^ concentration in the tissue of plant cells may function in inhibitory signal induction. Thus, this model defines root branching, as modulated by inhibitory signals via internal N status and external NO_3_^−^ supply [83].

Furthermore, ABA, which is associated with the systematic inhibitory effect of high NO_3_^−^ on LR growth, might be connected with the recently identified ABA receptor FLOWERING CONTROL LOCUS A (*FCA*). In addition, root architecture response to the recently identified external L-glutamate conceivably provides a significant tool for studying biological functions of plant glutamate receptors and amino acid signaling [98]. It was also reported that *FCA* possibly acts as a receptor for ABA. The loss of function mutant *fca* displays low sensitivity to the inhibitory effect of ABA on LRs, indicating that *FCA* might be a constituent in signaling transduction pathways associated with high NO_3_^−^ ABA-mediated inhibition of LRs [4,99,100].

It has been genetically proven that inhibition by ABA and NO_3_^−^ is mediated by the same signaling mechanism. For instance, the LABI (lateral roots ABA-insensitive) is characterized based on the LR production affinity when exposed to 0.5 µM, which is less sensitive to the high NO_3_^−^-induced LR inhibition [4] (Figure 3). The identification of *LABI* genes could give indepth information about the signaling mechanism underlying this inhibition [98]. Interestingly, all the mutants produced shorter primary roots phenotypes, which indicated that LR development could be intrinsically correlated with PR growth. It was reported that the presence of the PR meristem is required for high NO_3_^−^ and ABA-induced inhibition; however, this inhibition could be eliminated by the removal of the PRs [4].

Furthermore, root architecture response to glutamate may give an essential experimental framework to study glutamate signaling in plants and to elucidate the possible roles of the glutamate receptor [98]. Recent studies have shown that high NO_3_^−^ supply (30 mM) stimulated ABA accumulation in the emerging root tips by discharging it from the inactive stores via ER-localized β-GLUCOSIDASE1 (*BG1*) to regulate root development. This information provides a system for NO_3_^−^-induced root development via the regulation of ABA accumulation in the root tips. It was hypothesized that there is a close association between ABA and NO_3_^−^ signaling to coregulate LR growth [81]. A recent study has also shown that *myb29-1* mutants increased the LR length, LR density, and total length under adequate NO_3_^−^ supply in a genotype-dependent manner [48] (Table 1).

## 5. Coordinated Regulation of Nitrate and Other Messengers on RSA

Root foraging for NO_3_^−^ involves both local and systemic signaling. NO_3_^−^-auxin-CK regulation could also be a key constituent of N systemic signaling, which coordinates nutritional requirements among various organs at different growth stages [101,102].

### 5.1. Nitrate-Mediated Auxin Allocation

A systemic regulation that includes the inhibition of auxin translocation from the shoot to root suppresses LR initiation and development and subsequently affects NO_3_^−^ use efficiency in plants [103]. In such a situation, growing *Arabidopsis thaliana* on a nitrate medium was observed to have reduced auxin contents in the roots, while increasing the auxin content in the shoots. These findings have demonstrated that high NO_3_^−^ inhibits the translocation of auxin from the shoots to the roots [78].

In addition, nitric oxide (NO) was found to be a key nitrate-related signal that regulates plant RSA and the signaling cascade of lateral root formation induced by auxin [104]. It can be deduced from the previous observation that a decrease in NO_3_^−^ provision tends to promote auxin translocation from shoot to root. The high NO_3_^−^-inhibited root growth is a consequence of condensed cell elongation, and also probably due to the changes in meristematic length. Higher NO_3_^−^ supply diminished the IAA concentration in the phloem exudates. The NO_3_^−^-induced inhibited root growth was closely associated with the reduction of auxin in the roots, especially in the regions close to the root tips. The regrowth of PRs by external *NAA* and *IAA* under high NO_3_^−^ levels confirms that this inhibitory effect via high NO_3_^−^ might be partially associated with the reduced IAA level in the roots [42].

However, the effect of NO_3_^−^ on root growth could be complicated by the fact that high NO_3_^−^ concentration (50 mM) triggers complete inhibition of LR development [105]. It has been experimentally confirmed that these responses are linked to an auxin transport inhibitor. To this end, the local supply of nitrate reduced the transport of auxin from shoot to root, and this subsequently resulted in decreased root auxin concentration to a level more appropriate for lateral root growth. However, for the stimulation of LRs, a change in the root auxin concentration only is not adequate. Regardless of these models, few ideas concerning the transcriptional gene regulatory system are known [106].

Furthermore, under available nitrate conditions, the auxin level in the root decreased compared to low NO_3_^−^ conditions, and nitrate application seemed to inhibit auxin transport from shoot to root. In many cases, the external IAA partially lowers the stimulatory effect of localized nitrate. High nitrate supply reduces the IAA concentration in the phloem exudates; thus, suppression of root growth by high nitrate is mainly dependent on the reduction of IAA levels in the roots, specifically in the root tip region. It could be deduced that the inhibitory impact of high nitrate concentration on the restricted root growth may be associated with the decline in auxin content in the roots [42].

The currently accessible information leading to a potential connection between nitrate and auxin accumulation influences the rate of auxin biosynthesis, transport, and allocation of auxin from root to shoot [107].

### 5.2. Nitrate-Mediated Cytokinin Allocation

Cytokinin (CK) affects intercellular auxin transport by regulating the expression of numerous auxin transport components, and thus balances the auxin distribution to regulate the size of root meristem [108]. Findings have also shown that the NO_3_^−^-CK shoot–root dependent system exhibits the NO_3_^−^ demands of the whole plant, which affects root growth in NO_3_^−^ rich patches of the soil [109]. Since CK could be widely distributed throughout the entire plant cell, CK-induced root–shoot coordination is a proposed model of systemic signaling for nutritional status [110]. CK activity could be closely associated with NO_3_^−^ accessibility. Apart from the downstream metabolites of NO_3_^−^, NO_3_^−^ has been known to initiate rapid de novo CK synthesis and accumulation in *Arabidopsis* roots [111]. The CK biosynthesis occurs in different parts of the plant tissue, where the adenosine phosphate-isopentenyltransferase (IPT) is expressed. IPTs are the primary enzymes that mainly influence the rate of CK biosynthesis, such as the prenylation of adenosine 5′ phosphates and ATP and ADP at the N^6^- terminal with dimethyl diphosphate (DMAPP) [112].

In *Arabidopsis*, *IPT3* is regulated in a NO_3_^−^-dependent manner. The expression of *IPT3* with several Arabidopsis response regulators 3, 5, 6 (*ARR3, 5, 6*) are induced by NO_3_^−^ during the PNR. Moreover, *IPT3* is highly induced in the roots and weakly induced in the shoots in both WT and NR-null mutant plants during the PNR, partially mediated by *NRT1.1* [35]. During the PNR, *NIA* is among the highly inducible genes; thus, NO_3_^−^ firmly controls CK biosynthesis via activation of *IPT3*. This indicates that *IPT3* is the fundamental determinant of short-term NO_3_^−^-dependent CK biosynthesis, specifically in the roots, in response to immediate variation in the soil NO_3_^−^ [111]. In addition, the type-A *ARR* genes, including *ARR3*, *5*, and *7*, similar to the CK metabolism genes, were found to respond to NO_3_^−^ but not to NH_4_^+^. CYTOKININ RESPONSE FACTORS (CRFs) which are also highly inducible by NO_3_^−^ [15], are known to be transcriptionally activated by CK and its disruption influences the basal expression of a significant number of CK-regulated genes, including type-A *ARRs*. CRFs are involved in promoting plant growth and leaf senescence [113]. The close regulation of the *CYP735A2* and *IPT3* by NO_3_^−^ could be a major factor shaping NO_3_^−^-dependent spatio-temporal CK distribution in plants, and also regulating root system architecture in response to several abiotic stresses [114]. In short, nitrate and two hormonal mediators, CK and its antagonistic partner, auxin, act in synergy to modulate CK biosynthesis for root development.

## 6. Role of NO_3_^−^ Transporters in Mitigating Plant Stress

Nitrate transporters are ultimately responsible for the absorption of NO_3_^−^ from the soil and translocation of NO_3_^−^ to various aerial parts of the plant [115]. NO_3_^−^ transporter *NRT1.1* acts as a positive growth regulator of vegetative and reproductive organs [116]. Studies have shown that *AtNRT1.1/AtNPF6.3/CHL1* might be involved in the tolerance of the plant to proton toxicity; further studies on *chl1* mutants, however, have revealed a reduced proton tolerance when compared with WT [117]. Moreover, the accumulation of sodium (Na^+^) in the plant was found to be defective on *npf6.3/nrt1.1* mutants, thus *npf6.3/nrt1.1* functions in drought tolerance in the presence of NO_3_^−^ [118]. The downregulation of *NRT1.5* and the upregulation of *NRT1.8* were observed in the root of the plant on exposure to cadmium (Cd^2+^). Thus, increased NO_3_^−^ accumulation in the root [119] indicates that *NRT1.1* and *NRG2* function downstream from *NRT1.1* to regulate Cd^2+^ stress and also to stimulate NO_3_^−^ distribution to the root [119]. *ATNPF7.3/ATNRT1.5* is highly expressed in the root and highly inducible by phosphate starvation. The *ATNRT1.5* mutant *atnrt1.5* exhibits longer PRs, with reduced LR density under Pi-deficient conditions, compared with WT. This is an indication that a reduction in the morphological variation by ethylene synthesis antagonizes CO_2_ [120].

In addition to the transporters stated earlier, *npf6.4* mutants exhibit increased resistance to polyamine [115]. *AtNPF2.12/AtNRT2.6* positively regulated seed abortion under NO_3_^−^-deficient conditions in *Arabidopsis* [121]. Moreover, *AtNPF2.5* and *AtNPF2.3* induced chloride (Cl^−^) efflux from *Arabidopsis* roots and subsequently contributed to NO_3_^−^ translocation [122]. *AtNRT1.8/ANPF7.2* tolerates Cd^2+^ and salt stress. However, its knock-out mutants exhibited sensitivity to abiotic stress [123]. *AtNPF3.1* transported ABA and GA (gibberellic acid) in vitro [124]. The interaction between NO_3_^−^- and NRT-mediated NO_3_^−^ uptakes on exposure to Pb in *Arabidopsis* via NRT-related mutants [125] demonstrates a new strategy for plant tolerance to lead (Pb) contamination [125].

Under low NO_3_^−^ conditions, an NRT2 member, *AtNRT2.1,* contributes to iHATS (inducible high-affinity transport system) and plays a crucial role in the RSA, while *AtNRT2.4* contributes to plant biomass production. *AtNRT2.5* also stimulates mature plants under NO_3_^−^-deficient conditions [126]. *ATNRT2.6* expression is induced after phytopathogenic bacterium inoculation. Hence, plants with low *NRT2.6* expression show lower tolerance to pathogenic attacks [127]. Interestingly, there is a correlation between *NRT2.6* expression and reactive oxygen species (ROS) accumulation in response to *E. amylovora* infection and treatments with the redox-active herbicide methyl viologen. This indicates a probable link between *NRT2.6* activity and the production of ROS response to biotic and abiotic stresses [127].

In the chloride channel family (CLC), *AtCLC* accumulates anions in the vacuole when stomata are open, and also facilitates anion release during stomatal closure in response to stress hormones like abscisic acid (ABA) [128]. In addition to the NO_3_^-^ transporter, the NO_3_^−^-associated transcription factor, phloem-mobile CEPD-like 2 (*CEPDL2*)-polypeptide contributes to NO_3_^−^ acquisition, along with *CEPD1* and *CEPD2,* which mediate root N status, and the loss of each of these three proteins severely impair N homeostasis in the plants. A similar study showed that shoots of the *CEPDL2/CEPD1/2* genotype characterize a high-affinity NO_3_^−^ uptake duration in the roots, thereby indicating a systematic regulation of root N acquisition [84]. ANTHOCYANIN PIGMENT1 (*PAP1)* and its homolog *PAP2/MYB90* were strongly stimulated by NO_3_^−^ [129]. Recent research has demonstrated that three LBDs regulate anthocyanin synthesis via repression of *PAP1* and *PAP2*. MYB and bHLH (basic helix-loop-helix) proteins form complexes with TTG1 (TRANSPARENT TESTA GLABRA1) WD40-repeat protein in *Arabidopsis* to modulate several other epidermal gene expressions such as anthocyanin regulation, proanthocyanin, and mucilage biosynthesis in the seed coat or trichome and root hair organogenesis [49].

## 7. Conclusions

RSA response of the plant to NO_3_^−^ accessibility represents a prominent model to study developmental plasticity; however, the underlying mechanism remains highly obscure [130]. One of the most important discoveries in the past few years has been the involvement of NO_3_^−^ transporters *NRT1.1* and *NRT2.1* in early response signaling, and their effects on the morphological adaptation of the plant RSA. Despite their roles as transporters and in signaling response, NRT1.1 cannot fully explain the complete mechanism of the NO_3_^−^ responses observed in plants [43]. However, some findings have supported the previous speculation that NO_3_^−^ transporters could act as early NO_3_^−^ sensors [98]. This provides critical insights into understanding the ability to sense NO_3_^−^ as well as other nutrients [52].

In this review, we have summarized in depth the characterization of the nitrate transporters *NRT1.1* and *NRT2.1* in *Arabidopsis* (Figure 1), delivering clues on how NO_3_^−^ is sensed, taken up, and mobilized, and their modification by phosphorylation at the T101 residue has also been well demonstrated. In addition, the influences of physiological growth on RSA under low and high NO_3_^−^ conditions, and the underlying molecular players, including TFs and N metabolites, are hypothesized and are associated with the transcriptional control of significant NO_3_^−^-responsive genes, which include *NIA1*, *NIA2*, *NiR*, *NR*, *NRT2.1*, -*2.2*, -*2.4*, -*2.5*, and *NRT3.1*. However, the fact is that different TFs, *NLP7*, *TGA1/4*, and *TCP20,* can regulate the expression of the same target gene, *NRT2.1* (Figure 1). These TFs co-interact in response to NO_3_^−^ to regulate root growth.

Despite the development of multiple NO_3_^−^ signaling pathways regulating RSA and the characterization of primary Ca^2+^-induced responses elucidated in the present review, many important inquires on how PLCs are implicated in nitrate signaling and the specificity of the protein kinases that switch the different constituents of PLCs are yet to be answered. Moreover, the speculated nonphosphorylated form of the NRT1.1-signaling Ca^2+^-CPKs-NLPs pathway has received trivial experimental attention. Additionally, PLC- and Ca^2+^-independent nitrate signaling pathways have another component, as evidenced by *AFB3* expression and its downstream TFs, which lead to the possibility that there might be another second messenger involved in nitrate responses.

There are more nitrate regulatory modules in existence, with no clues about their signaling pathways and components; however, they are the fundamental contributors controlling LR development. Hence, functional identification and characterization of the various players associated with this and other NO_3_^−^ signaling pathways and their possible functions in the root architecture of *Arabidopsis* is the next step to try and comprehend the NO_3_^−^ responses that will facilitate crop genetics improvement.

## Figures and Tables

**Table 1:** Transcription factors of genes associated with nitrogen signaling and nitrogen-associated processes in *Arabidopsis thaliana.*

Transcription Factors	Family	Transcriptionally Associated with NO^3−^ Signalling	Tissue Expression	Molecular Function	Effect on Root	Localization	Refs
*CEPD2*	CC-type glutaredoxin (ROXY) family	yes	Root, root endodermis, root vascular system	Cellular response to nitrogen starvation	Regulate the efficiency of root N acquisition	cytoplasm, nucleus	[84]
*AtGRXS3/4/5/8/ROXY11*	CC-type glutaredoxin (ROXY) family	yes	Root and other tissue	Cell redox homeostasis	Increased primary root length	cytoplasm, nucleus	[85]
*ERF4*	Subfamily B-1 of ERF/AP2 transcription factor family	yes	Root and other tissue	Transcription regulatory region DNA binding	Antagonizes JA inhibition of root elongation	nuclear body, nucleus	[48,86]
*RAV2*	Ethylene-responsive element-binding protein family	yes	Root and other tissue	Transcription regulatory region DNA binding	Genotype based Shorter LRL to both high and low NO^3−^	nucleus	[48]
*VIP1*	VIRE2-interacting protein 1	yes	Root and other tissue		unknown	cytosol, nucleus	[48]
*ERF070*	Ethylene-responsive element-binding protein family	yes	Root and other tissue	Regulation of transcription	unknown	nucleus	[48]
*HMGB15*	AT-rich interaction domain-containing transcription factor family	yes	Root and other tissue	Glucosinolate metabolic process,	Larger LRs response to nitrate deprivation	nucleus, pollen tube	[48]
*PAP2/MYB90*	MYB domain transcription factor family	yes	unknown	Regulation of transcription,	Trichome and root hair organogenesis	nucleus	[49,87]
*BBX16*	Constans-like zinc finger family	yes	unknown	Positive regulation of transcription	Total LRs length (LRL)	nucleolus, nucleus	[48]

## Abbreviations

NRT1	Nitrate transporter1
CLC	Chloride Channel
CIPK	CBL-Interacting Protein Kinase
FIP1	Factor interacting with poly (A) polymerase 1
CPSF30-L	Cleavage and Polyadenylation Specificity Factor 30-L
ABI2	ABA-insensitive 2
AFB3	AUXIN RECEPTOR F-BOX3
NAC4	NAC-domain containing protein 4
OBP4	OBF Binding Protein 4
TGA1/4	Targets the activation sequence1/4
EGTA	Ethylene glycol tetraacetic acid
CEP	C- TERMINALLY ENCODED PEPTIDE
LaCl_3_	Lanthanum (III) chloride hydrate
PI-PLC	Phosphatidylinositol-specific phospholipase C
TCP20	Teosinte branched1/cycloidea/proliferating cell factor1-20
ANR1	ARABIDOPSIS NITRATE REGULATED1
ARF	AUXIN RESPONSE FACTOR
SCFTIR/AFB	S-PHASE KINASE ASSOCIATED PROTEIN 1-CULLIN-F-BOX PROTEIN (SCF)-type E3 ubiquitin-protein ligases
MEKK1	Mitogen-Activated Protein Kinase Kinase 1
BBX	Bobby sox homolog
ROXY	Floral glutaredoxins
CEPD	Phloem-specific peptides
ERFs	Ethylene-responsive element binding factors
RAV2	Regulator of V-ATPase in vacuolar membrane protein 2
